# IodiNe Subtraction mapping in the diagnosis of Pulmonary chronIc thRomboEmbolic disease (INSPIRE): Rationale and methodology of a cross-sectional observational diagnostic study

**DOI:** 10.1016/j.conctc.2019.100417

**Published:** 2019-07-24

**Authors:** Yousef Shahin, Christopher Johns, Kavitasagary Karunasaagarar, David G. Kiely, Andy J. Swift

**Affiliations:** a Department of Infection, Immunity and Cardiovascular Disease, University of Sheffield, Sheffield, UK; b Department of Clinical Radiology, Sheffield Teaching Hospitals, Sheffield, UK; c Sheffield Pulmonary Vascular Disease Unit, Royal Hallamshire Hospital, Sheffield, UK; d INSIGNEO, Institute for in Silico Medicine, University of Sheffield, UK

**Keywords:** INSPIRE, Pulmonary embolism, Chronic thromboembolic disease, Iodine subtraction, CTLSIM

## Abstract

Chronic thromboembolic pulmonary hypertension (CTEPH) is a severe but treatable disease that is commonly underdiagnosed. Computed tomography lung subtraction iodine mapping (CT-LSIM) in addition to standard CT pulmonary angiography (CTPA) may improve the evaluation of suspected chronic pulmonary embolism and improve the diagnostic pick up rate. We aim to recruit 100 patients suspected of having CTEPH and perform CT-LSIM scans in addition to the current gold standard test of nuclear medicine test (lung single photon emission computed tomography (SPECT) imaging) as a pilot study which will contribute to and inform the definitive trial. The diagnostic accuracy of CT-LSIM and lung SPECT will be compared. The primary outcome of the full definitive study is non-inferiority of CT-LSIM versus lung SPECT imaging.

## 1 Introduction

Chronic thromboembolic pulmonary hypertension (CTEPH) is a treatable, life-threatening disease that occurs in up to 4% of patients following acute pulmonary embolism (PE) [1]. The disease is characterised by remodelling of the pulmonary arteries due to poor clearance of clot. Prognosis is very poor without treatment, and pulmonary endarterectomy (PEA) is well established as the definitive and potentially curative treatment method for CTEPH.

The European Society of Cardiology recommends ventilation/perfusion single photon emission tomography (V/Q SPECT) as the first line-screening test for patients with CTEPH. The perfusion image involves injection of 99mTc labelled macroaggregated human albumin, exposing the patient to ionizing radiation and the study acquisition time is 30–40 min.

Evaluation of the pulmonary arterial tree by computed tomography pulmonary angiography (CTPA) and lung perfusion is required to determine the appropriate treatment strategy in chronic thromboembolic disease (CTED). Recently, there has been much interest in the application of lung perfused blood volume images using dual-energy CT (DECT) to assess lung perfusion [2,3]. However, DECT is not widely available in hospitals across the UK and V/Q SPECT remains the reference standard. The rationale, methodology and design of the IodiNe Subtraction mapping in the diagnosis of Pulmonary chronIc thRomboEmbolic disease (INSPIRE) study are summarised in this paper.

### 1.1 Rationale of the INSPIRE study

Computed tomography lung subtraction iodine mapping (CT-LSIM) and accompanying software is now available in routine clinical practice (Sure subtractionTM, Toshiba Medical Systems; FDA report K130960). CT-LSIM images are created using on a non-rigid registration of a low dose unenhanced thoracic CT to a CTPA, with both examinations performed during the same sitting in less than 10 min total scanning time. Subtraction of the non-contrast CT from the contrast-enhanced CTPA produces the CT-LSIM. CT-LSIM simultaneously provide high-spatial-resolution images of the pulmonary arterial tree and parenchymal anatomy in combination with functional examination of lung perfusion.

Magnetic resonance imaging (MRI) is an alternative approach with the advantage of the lack of ionizing radiation and can produce lung perfusion maps with good diagnostic accuracy for CTED [5]. MRI is relatively limited in comparison to CT in terms of availability and the lack of ability to provide an out of hours service in some centres. Recently, it has been shown that Gadolinium is deposited in the basal ganglia, the clinical significance of the retained gadolinium in the brain, if any, remains unknown [4]. Further research is ongoing.

A recent meta-analysis and systematic review, highlights the diagnostic potential of CT in both screening and for surgical and interventional operability [6].

Replacement of CT for V/Q SPECT in the setting of screening for CTED would lead to a £325 saving per patient. The diagnosed annual incidence of CTEPH is approximately 700 cases in the UK, projected to rise to about 1000 in 2025. Estimated pick up rate of perfusion defects in patients with suspected CTED is 59% at a specialist centre (pick up rates are likely to be much lower at non-specialist centres). An estimated 1186 patients are screened at specialist centres, if these patients were screened using CT instead of SPECT, cost savings would be made in the UK based on current estimates.

In patients found to have CTED on lung SPECT, CTPA is also required to characterise the extent of pulmonary arterial clot for surgical planning which adds further cost. The total saving of a pathway using CTPA with iodine subtraction mapping for screening and surgical planning is potentially significant.

#### 1.1.1 Aims and objectives of the INSPIRE study

The main objective of the INSPIRE study is to assess the diagnostic performance of CT-LSIM for evaluation of pulmonary perfusion in patients with known or suspected CTEPH. The primary hypothesis is that CT-LSIM is more sensitive than V/Q SPECT and CTPA in the assessment of CTED in patients with known or suspected CTEPH. We also hypothesize that the diagnostic performance of the experienced observers will be better than the less experienced and that the number of alternate diagnoses made on CT-LSIM will be higher and more accurate than lung SPECT imaging.

## 2 Materials and methods

### 2.1 Design and setting

The INSPIRE study is a single centre cross sectional observational diagnostic study comparing the diagnostic accuracy for new test CT-LSIM with nuclear medicine SPECT. The setting of the study is a tertiary referral centre for pulmonary hypertension. Patients will be recruited during their admission to the Sheffield Pulmonary Vascular Disease unit. CT scans will be performed in the Radiology Department at the Royal Hallamshire Hospital. Patients will be identified by the Pulmonary Hypertension team from their clinic visit as suspected CTED or CTEPH. Patients will be sent information sheets and written informed consent and those will be obtained from all the patients. Consent will be taken by a research team member on admission for diagnostic visit that includes CT scanning. The study is registered on clinicaltrials.gov (NCT03806907).

### 2.2 Eligibility

Patients are eligible to be included in the INSPIRE study if they are suspected to have CTED or CTEPH and require a SPECT and CTPA.

Patients will be excluded if they are less than 18 years old, unable to provide informed consent, have significant renal dysfunction (GFR <30 ml/min), have a history of hypersensitivity to contrast material or pregnant.

### 2.3 Imaging protocol

Images will be evaluated for objective and subjective image quality. No additional contrast will be used as compared to standard clinical practice as patients will only undergo one CTPA scan. The CT protocol of this study has been carefully designed to have a radiation dose identical or even lower than standard CT protocols for PE detection.

### 2.4 CTPA and LSIM acquisition

CT examinations will be performed on a 320 detector-row CT system (Aquilion ONE/ViSION edition; Toshiba Medical Systems, Otawara, Japan). After non-contrast CT is performed, contrast material will be injected for 25 s via an antecubital vein using a weight-adapted injection protocol. Scanning will be initiated 3 and 14 s after the attenuation in the region of interest (ROI) placed in the pulmonary artery reached the threshold of +100 HU under the single breath hold. CT images will be reconstructed using adaptive iterative dose reduction.

The image data sets shall be analyzed with the algorithm on the post-processing software attached to Aquilion ONE/Vision Edition (SURESubtraction Lung). Based on a non-rigid registration followed by subtraction of the non-contrast images from the contrast-enhanced images (CTPA images), a colour map is produced showing the iodine distribution in the lung parenchyma.

### 2.5 Nuclear medicine SPECT acquisition

SPECT imaging will be performed on a General Electric Infinia SPECT system using a low energy general purpose collimator. 100 MBq 99mTc MAA will be administered through a direct intravenous injection with a needle of 21G or larger. The image acquisition parameters are: acquisition matrix 128x128, 60 projections per detector and 7 s per projection. Images will be acquired prone with the patient's arms extended above their heads, where possible.

**Fig. 1:**
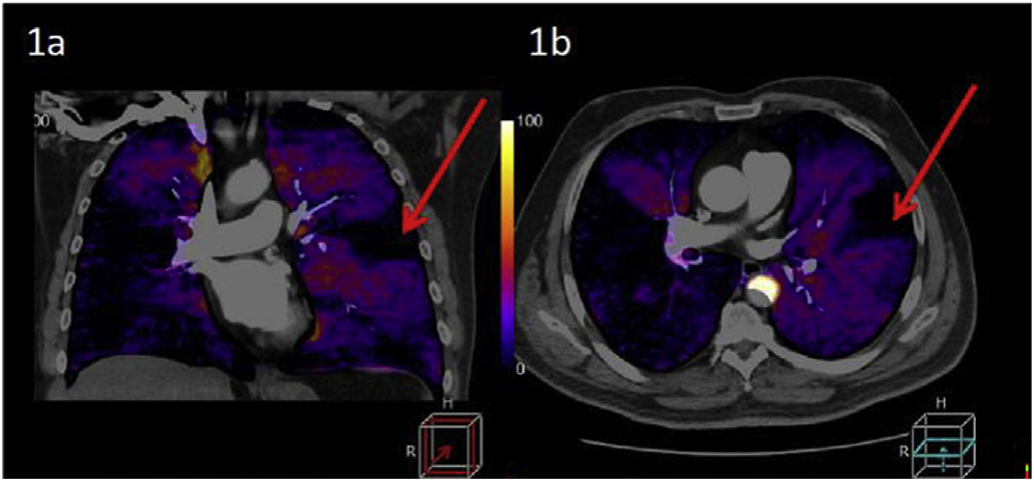
Coronal (1a) and axial (1b) CT-LSIM showing a classical triangular segmental perfusion defect (red arrows). (For interpretation of the references to colour in this figure legend, the reader is referred to the Web version of this article.)

**Fig. 2:**
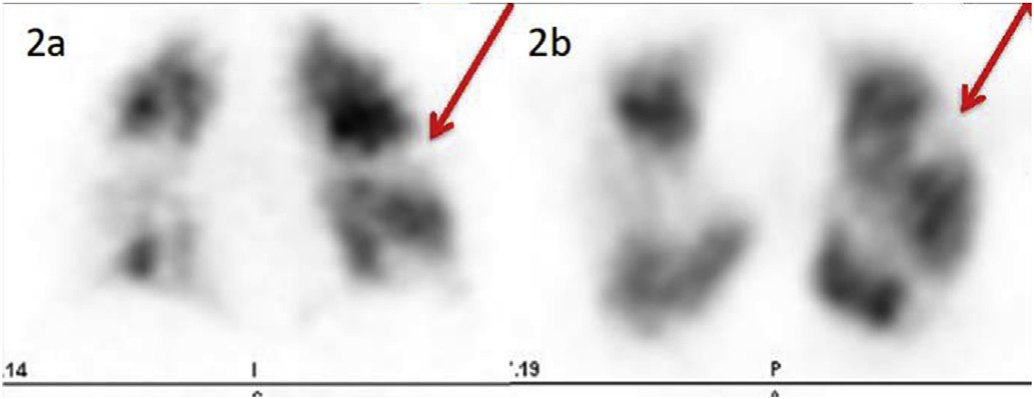
Coronal (2a) and axial (2b) SPECT lung perfusion scintigraphy images showing the same segmental perfusion defect. Note the sharper delineation of the perfusion defect on CT.

Fig. 1 illustrates coronal (1a) and axial (1b) CT-LSIM showing a classical triangular segmental perfusion defect (red arrows). Fig. 2 illustrates coronal (2a) and axial (2b) SPECT lung perfusion scintigraphy images showing the same segmental perfusion defect. Note the sharper delineation of the perfusion defect on CT.

### 2.6 Radiation dose

The typical effective dose for lung perfusion scintigraphy, was 2.04–2.86 mSv determined by the normalized coefficient. A study of 50 patients [7] using the same scanner used in our hospital found a median dose length product of 171 mGy cm, corresponding to an effective radiation dose of approximately 2.90 mSv, using a conversion factor of 0.017 mSv/mGycm for chest CT. Hence the advantages described above can be achieved with a similar radiation dose to a nuclear medicine lung scintigraphy scan and patients who require both CTPA and SPECT have a lower total dose.

### 2.7 Image analyses

All images will be anonymised and given unique study identifiers. Analysis will be performed using a commercially available diagnostic workstation (OsiriX).

### 2.8 Radiological evaluation

CT-LSIM and CTPA images shall be respectively evaluated by three observers, two consultant Radiologists and a Radiology resident who were blinded to the clinical data (observer 1: a thoracic radiologist with 8 years of specialist experience, observer 2: a thoracic radiologist with 7 years of experience and observer 3 a Radiology resident with 4 years’ experience), following which a consensus read will be performed by observer 1 and 2. The scores of each reader will be adopted, as they were if scores are matched. If scores of each reader are different, either shall be adopted after the discussion of two readers. In addition, the 2nd reading session of observer 1 will be performed to determine intra-observer agreement.

LSIM images will be scored primarily as positive, negative or indeterminate. If abnormal a severity score will be assigned. Score 1, if normal perfusion, score 2 if mild perfusion defects (<25%); score 2, moderate perfusion defects (25–50%), and class 3, severe perfusion defects (>50%).

CTPA will be scored primarily as positive, negative or indeterminate. In addition, the clot burden will be semi-quantitatively scored as mild, moderate or severe, and predominantly proximal or distal.

### 2.9 Sample size

This study has been powered to demonstrate non-inferiority of CT LSIM compared to SPECT lung scintigraphy, with 90% power, assuming a percentage success estimated from a previous study, assuming 95% sensitivity and 95% specificity; a 10% non-inferiority margin and 11% discordant pairs. This proportion must be bigger than the non-inferiority margin of 10%. With 90% power and 5% two-sided significance and estimated prevalence of 60%, 290 cases are required (116 without the disease and 174 with the disease). The pilot study we will aim to recruit 100 cases which will contribute to the definitive trial. The analysis of the results of the pilot study will allow a more precise estimate of full trial size.

**Fig. 3:**
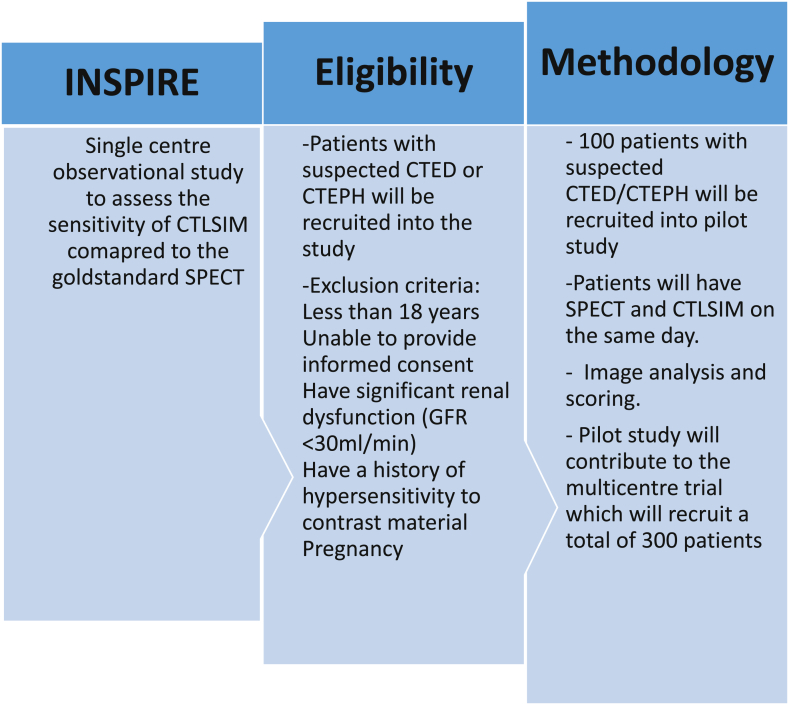
Flow chart diagram of study design. CTED; chronic thromboembolic pulmonary disease, CTEPH; chronic thromboembolic pulmonary hypertension.

We perform approximately 260 SPECT lung scintigraphy scans a year for suspected CTED/CTEPH and we expect to recruit 50% of these patients based on studies on CTED/CTEPH patients previously done at our institution. Hence would expect to complete the pilot study recruitment (n = 100) in 1 year and the full study in approximately 3 years depending on recruitment rate and sensitivity/specificity results from the pilot study. A flow chart of study design is shown in Fig. 3.

### 2.10 Statistical analysis

A Statistical Package for the Social Sciences Program (SPSS) version 24 for Windows (SPSS Inc. Chicago, IL) will be used for statistical analysis. Continuous variables will be expressed as mean ± standard deviation (SD) for parametric variables and median (interquartile range) for non-parametric variables. Categorical data will be presented as the number of subjects and percentage.

A Chi-squared test will be used to establish sensitivity, specificity, positive and negative predictive values for detecting the presence and absence of PE. The Kappa (*κ*) statistic will be used to determine the level of agreement between the independent observers. The *κ* value will be defined as follows: values < 0 indicated lack of agreement; 0–0.20, slight agreement; 0.21–0.40, fair agreement; 0.41–0.60, moderate agreement; 0.61–0.80, substantial agreement; 0.81–1, almost perfect agreement.

All statistical tests will be two sided with a p value of less than 0.05 considered to be statistically significant.

## 3 Discussion

The INSPIRE study is a single centre prospective observational study which will aim to assess the sensitivity and specificity of CT-LSIM compared to CTPA and SPECT imaging in the diagnosis of CTEPH. It will also assess the performance of CT-LSIM in the evaluation of lung perfusion compared to the previous imaging modalities in CTEPH patients.

INSPIRE is the first study to compare the specificity and sensitivity of CT-LSIM to CTPA and SPECT imaging in the diagnosis of CTEPH. The only other study which assessed the diagnostic performance of CT-LSIM was conducted by Tamura et al. [7] comparing the diagnostic performance of CT-LSIM to CTPA using SPECT as a reference standard in 50 patients with CTEPH. The authors concluded that CT-LSIM is a feasible technique for the assessment of segmental lung perfusion in patients with CTEPH and provides a higher diagnostic accuracy compared to CTPA. Of note, Tamura et al. [7] only compared CT-LSIM to CTPA. Furthermore, SPECT imaging was based on lung perfusion only with no assessment of ventilation. The sample size is also small to draw any generalizable conclusions which was acknowledged as a limitation of the study. INSPIRE is the first study to provide a head to head comparison of CT-LSIM with SPECT which is the current gold standard in a large cohort of patients. The aim of the INSPIRE study is to become a multicentre study following the initial single centre pilot phase and recruit approximately 300 patients who are diagnosed or suspected to have CTEPH.

Previous studies have compared dual energy CT (DECT) [[8], [9], [10]], CTPA [11] and 3-D contrast enhanced lung perfusion MRI [12] to SPECT imaging for the evaluation of segmental lung perfusion in patients with CTEPH. These studies showed superior diagnostic performance of DECT compared with SPECT but not CTPA. However, despite DECT proving to be superior to SPECT imaging, the latter remains the reference standard perhaps due to the small number of patients included in these studies and because of lack of access to DECT due to the high cost.

CTPA, although offers an improved better spatial resolution and additional morphological information compared to nuclear medicine scintigraphy, it has lower sensitivity in detecting peripherally located perfusion defects compared to SPECT. Thus, SPECT imaging remains superior to CTPA despite the latter showing higher specificity [13].

CTEPH is a life shortening complication of acute PE which results in the formation of intravascular scars leading to increased pulmonary hypertension [14]. Currently, the gold standard treatment for these lesions is PEA in patients deemed fit for surgery and for proximal disease [15]. For patients who are unfit for surgery and for lesions which are inaccessible surgically such as segmental and subsegmental disease, balloon pulmonary angioplasty (BPA) has been recently introduced as a treatment option [16,17]. Therefore, pre BPA planning of segmental perfusion is essential and currently is assessed by a combination of CTPA and SPECT. INSPIRE hypothesise that CT-LSIM has the potential to offer a more accurate visualisation and assessment of lung segmental perfusion as pre-treatment planning tool for patients eligible for BPA.

The results of this study are important as CT-LSIM has the potential to cut down the cost of having two investigations (CTPA and SPECT), reduce the radiation dose by approximately 3–4 mSV and can be a one stop investigation reducing the number of patients visits to hospital from two to one visit.

## Funding

This study is funded by a Wellcome Foundation Trust Fellowship award to Dr Andy Swift and a pump priming Academic Clinical Lecturer grant to Dr Yousef Shahin from the University of Sheffield.
